# Stem cell therapy as a promising approach for ischemic stroke treatment

**DOI:** 10.1016/j.crphar.2024.100183

**Published:** 2024-05-16

**Authors:** Sahar Yaqubi, Mohammad Karimian

**Affiliations:** Department of Molecular and Cell Biology, Faculty of Basic Sciences, University of Mazandaran, Babolsar, Iran

**Keywords:** Ischemic stroke, Neuron, Chemical drugs, Stem cell therapy

## Abstract

Ischemia as the most common type of stroke is the main cause of death and disability in the world. However, there are few therapeutic approaches to treat ischemic stroke. The common approach to the treatment of ischemia includes surgery-cum-chemical drugs. Surgery and chemical drugs are used to remove blood clots to prevent the deterioration of the nervous system. Given the surgical hazards and the challenges associated with chemical drugs, these cannot be considered safe approaches to the treatment of brain ischemia. Besides surgery-cum-chemical drugs, different types of stem cells including mesenchymal stem cells and neurological stem cells have been considered to treat ischemic stroke. Therapeutic approaches utilizing stem cells to treat strokes are promising because of their neuroprotective and regenerative benefits. However, the mechanisms by which the transplanted stem cells perform their precisely actions are unknown. The purpose of this study is to critically review stem cell-based therapeutic approaches for ischemia along with related challenges.

## Highlights

• Conventional stroke treatment integrates surgery and pharmaceutical interventions.
• Stem cell therapies emerge as a promising avenue in stroke treatment.
• Safety remains a paramount challenge in the realm of stem cell therapy for stroke.

## Graphical abstract

The mechanism of the function of the tPA for the treatment of ischemic stroke and its risks. With the cleavage of plasminogen, the tPA produces the active enzyme of the plasmin, which in turn dissolves fibrin-based clots in ischemic stroke. When given to patients, the tPA effectively causes ischemic brain reperfusion. Increased tPA proteolytic activity results in increasing edema and inflammatory signals, and destruction of the blood barrier which makes the consequences of stroke more complicated. Also, tPA administration, especially its delayed prescription, is associated with increased intra-skull bleeding, hemorrhagic transformation and death.

**Figure:**
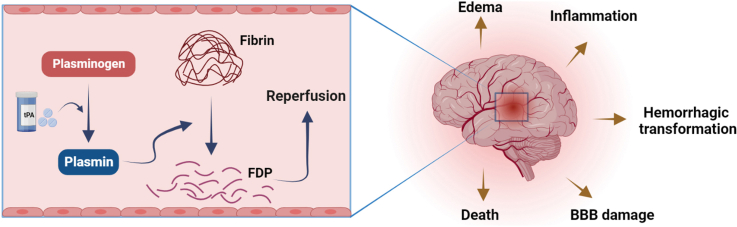

**Table:** Abbreviations

BDNF	Brain-derived neurotrophic factor
BLI	Bioluminescence imaging
ESCs	Embryonic stem cells
EVs	Extracellular vesicles
HA	Hyaluronic acid
IHF	Ischemic heart failure
ILs	Interleukins
iPSCs	Inducible pluripotent stem cells
MCAO	Middle cerebral artery occlusion
MRI	Magnetic resonance imaging
MSCs	Mesenchymal stem cells
NSCs	Neural stem cells
NSPC	Neural stem/progenitor cells
PAIS	Perinatal arterial ischemic stroke
pDDIs	potential Drug-Drug Interactions
PET	Positron emission tomography
SPECT	Single-photon emission computed tomography
tPA	Tissue plasminogen activator

## 1 Introduction

Stroke as a multi-factorial disorder is the second most common cause of mortality and the major cause of long-term disability. One-third of stroke victims suffer from functional and neurological disorders following the incident (Boehme et al., 2017). Stroke is the result of obstruction or rupture of blood vessels, and almost more than 80% of all stroke cases are ischemic, which are usually caused by blockage of the middle cerebral artery (Kuriakose and Xiao, 2020; Tajalli-Nezhad et al., 2019; Vahidinia et al., 2021). Researchers have demonstrated that risk factors such as age, gender, hypertension, smoking, unhealthy diet, physical inactivity, diabetes, excessive alcohol and drug use, heart diseases as well as genetic factors are all associated with the risk of ischemic stroke (Boehme et al., 2017; Nejati et al., 2018). The more common approaches for the treatment of brain ischemia include surgery-cum-chemical drugs (Behdarvandy et al., 2020; Zhu et al., 2018).

The main purpose of treating brain ischemia with chemical and surgical drugs is to remove blood clots to prevent the deterioration of the nervous system (Nozohouri et al., 2020; Tameh et al., 2018). Given the surgical hazards, it is not generally regarded as a safe option in the treatment of brain ischemia. In addition to surgery, the use of FAD-approved chemical drugs can be effective for the treatment of ischemic stroke (Nozohouri et al., 2020; Vahidinia et al., 2020). Medicinal options for the treatment of ischemic stroke include tissue plasminogen activator (tPA), aspirin, warfarin, clopidogrel and statins (Tremonti and Thieben, 2021). Despite the efficiency of chemical drugs in the treatment of ischemic stroke, some restrictions are considered for their application. For instance, they should be initiated in patients with clinical standards shortly after the development of ischemic stroke (Brzica et al., 2017; Khassafi et al., 2022; Vahidinia et al., 2022). There are also challenges such as the potential of drug-drug interactions, difficulty getting the drug through blood-brain barrier, gastric bleeding, etc. in the course of treatment of ischemic stroke with chemical drugs (Aleksic et al., 2019; Hochain et al., 2000; Pardridge, 2001). While antiplatelet treatment with aspirin diminishes the risk of early stroke recurrence within 48 h following its onset, it does not the ability to treat the previously formed stroke. Newer antiplatelet agents alone or combined with aspirin, demonstrated promising results for prevention of premature recurrence of stoke, though clinical tests are still underway (Bansal et al., 2013).

Numerous studies have been dedicated to evaluate the therapeutic potential of various types of stem cells including mesenchymal stem cells, inducible pluripotent stem cells, embryonic stem cells, and neural stem cells as a therapeutic option for the treatment of ischemic stroke (Marei et al., 2018). Stem cell-based therapies for stroke are promising solutions due to their neuroprotective and regenerative potential (Mahla, 2016). The aim of stem cell-based therapies for the treatment of stroke is neurological regeneration, diminished loss of neurons and neuronal protection in the acute phase of stroke to limit the spread of injury (F. Wang et al., 2018). The results of stroke treatment with stem cells are interesting, but many have provided contradictory results. Besides, the underlying mechanisms by that transplanted stem/precursor cells perform their actions are largely unknown (Misra et al., 2012). The purpose of this study is to describe the stem cell-based therapeutic approaches for ischemia along with the challenges facing them.

## 2 Common therapies for ischemic stroke: surgical procedures, pharmacological treatments, and their associated challenges and complications

In recent years, physicians have resorted to chemical drugs and surgery to treat ischemic stroke. The main goal of ischemic stroke treatment with the above-mentioned methods is to prevent blood clots from advancement in order to prevent the deterioration of the nervous system (Barahimi et al., 2021; Nozohouri et al., 2020). The aim of endovascular thrombectomy is to be the preferred therapeutic strategy for the treatment of acute ischemic stroke induced by the occlusion of large cerebral arteries, mainly in cases with high clot burden or in the presence of contraindications of intravenous thrombolysis (Berkhemer et al., 2015). Prompt cannulation of occluded vessels is critical for obtaining a good clinical outcome (Khatri et al., 2009). Carotid arteries are most often accessible through femoral puncture and catheterization of aortic arch within minutes. However, there are some conditions in which this approach becomes impossible or time-consuming with negative impacts on clinical outcomes (Levy et al., 2002).

Considering the risks of surgery, it cannot be considered as a safe approach for the treatment of ischemic stroke. Besides surgery, application of chemical drugs approved by FAD can be effective in the treatment of ischemic stroke (Nozohouri et al., 2020). Regarding enhancement of rehabilitation following stroke, numerous drugs have demonstrated promising results in small clinical trials, however, none of them have been assessed in large phase III clinical trials or approved by US Clinical Supervision. Some agents such as tenecteplase, edaravone, and minocycline may be approved for universal use in the future (Kikuchi et al., 2014).

Drug treatment for stroke can be divided into categories of specific stroke treatment and stroke prevention (Gilman, 2006). Available drug options for the treatment of ischemic stroke include tissue plasminogen activator and antiplatelet agents. The major role of medical treatment of stroke is to control the patient's blood pressure and intracranial pressure (Hinkle and Guanci, 2007). Currently, some drugs are thought to act on neurotransmitters or neuromodulators in the central nervous system, while others appear to act at peripheral neuromuscular sites. In addition, each drug agent has the potential for having side effects that should be considered before prescribing (Gallichio, 2004). The development of agents with the potential to diminish brain damage subsequent to the development of ischemic stroke requires novel and diverse approaches based on a better understanding of underlying pathophysiologic mechanisms of ischemic stroke. Furthermore, the future therapeutic strategies for ischemic stroke are possibly combined therapy rather than monotherapy. Besides, additional approaches for testing and applying neurovascular protectors should also be considered (Kikuchi et al., 2014).

Despite the efficacy of pharmacotherapy in the treatment of ischemic stroke, several limitations have been reported regarding their use. For example, they should be prescribed in a short period of time following stroke onset in patients who meet the clinical criteria (Brzica et al., 2017). Another problem is the phenomenon of potential Drug-Drug Interactions (pDDIs). This happens in the setting of altered impacts of one drug by other concomitantly applied drug(s). The appearance of pDDIs in cases who suffered from acute ischemic stroke was most related to the total number of drugs prescribed, length of hospitalization and older age (Aleksic et al., 2019). Indeed, the brain is physiologically different from other organs. Blood vessels have a strong endothelial layer, and blood-brain barrier has made it difficult for drugs to pass through it. The blood-brain barrier protects the brain from other toxins and infections but is one of the biggest barriers to drug delivery, too (Pardridge, 2001).

## 3 Challenges in categorizing stem cells as drugs

Stem cells, as a therapeutic method, represent a paradigm shift in medicine that offers revolutionary potential in the treatment of various diseases and injuries (J. Wang et al., 2024). Stem cells are unique in their ability to self-renew and differentiate into specialized cell types, making them valuable tools for regenerative medicine and tissue engineering (Jin et al., 2023). In the field of drug development and regulation, stem cells present unique challenges and considerations. Unlike traditional small molecule drugs that exert their effects through specific molecular interactions, stem cells are living organisms with complex biological properties (Mansour et al., 2023). Mechanisms of action of stem cells are multifaceted, involving processes such as cell differentiation, paracrine signaling, and immune modulation. In this way, the development and evaluation of stem cell therapies requires a different regulatory framework and approach compared to conventional drugs (George, 2011).

With all these attributes in the definition of stem cells as a drug or a tool, different ideas are raised. A drug is any substance, other than food, that produces a physiological change in the body when taken in various forms, such as inhalation, injection, smoking, consumption, absorption through an adhesive on the skin, or dissolution under the tongue (Jin et al., 2023). In pharmacology, drugs, also known as pharmaceutical drugs, are used to treat, cure, prevent, or diagnose diseases, as well as to promote well-being. According to this definition, drugs must meet certain criteria, including having an indication to treat various diseases and being available as off-the-shelf products (Rubin and Haston, 2011; Wittich et al., 2012). Therefore, according to this definition, stem cell drugs are defined as off-the-shelf products based on stem cells for the treatment, cure, prevention, or diagnosis of diseases, or the promotion of well-being (Van Pham, 2016).

Stem cell drugs, as off-the-shelf products are used in allogeneic stem cell transplantation. There are important differences between allogeneic stem cell transplants and stem cell drugs. The main difference is that stem cell drugs are products, while allogeneic stem cell transplantation is a method of using these drugs. Furthermore, the former is approved as a drug, while the latter is approved as a medical device (Falkenburg et al., 2008). The defining characteristics of stem cell drugs include 1- Derivation from stem cells, 2- Readily available for use, 3- Manufactured in large quantities with consistent quality, 4- Subject to quality control measures by Good Manufacturing Practice guidelines, and 5- Officially approved as drugs. However, the features of allogeneic stem cell therapy encompass 1- The methodologies employed in cell utilization, 2- Utilization either as off-the-shelf products or directly from donors, 3- Variation in quality across batches based on donors and production methods, and 4- Approval as medical devices (Van Pham, 2016).

In recent years, regulatory agencies around the world have developed guidelines and regulations specifically for the development and evaluation of cell-based therapies, including stem cell products (Louria, 1969). The purpose of these regulations is to ensure the safety, efficacy, and quality of stem cell-based products while facilitating their translation from preclinical research to clinical practice. However, navigating the regulatory landscape for stem cell therapies remains complex and requires collaboration among researchers, clinicians, regulatory agencies, and industry stakeholders (Abubakar et al., 2023). The administration of stem cells in various diseases faces contradictions at different levels, including social acceptance, regulatory barriers, and scientific uncertainties. These contradictions highlight the need for balanced dialogue, informed decision-making, and sustainable settings to ensure responsible progress in medical science and technology (Rosen, 2006).

## 4 Utilizing stem cell therapy for ischemic stroke treatment

There is a growing interest in using stem cells for the treatment of numerous neurological diseases such as ischemic stroke (Leader et al., 2008). Several cell types are lost during the development of an ischemic stroke and repairment of blood vessels (pericytes, smooth muscle cells, and endothelial cells) as well as oligodendrocytes, neurons, and astrocytes is of paramount importance. The advantage of cell-based treatments and other restorative therapies is their ability to be used for longer periods of time (Bath and Sprigg, 2006). Stem cell-based therapy may be administered within days, weeks, or even months following injury, providing more benefits to patients (Hess and Borlongan, 2008).

Stem cell therapy has been considered in research fields as a promising regenerative therapy and a more promising therapeutic strategy for induced brain damage by various types of strokes. Stem cells, whether induced pluripotent stem cells or endogenous neural stem cells have the potential to replace damaged brain cells. Cell replacement strategies have been evaluated in numerous animal stroke models within decades of research (Caplan, 2007). Indeed, beneficial paracrine effects have been observed using pluripotent stem cells. Reduced cell death, facilitated growth/nutritional support for host cells and enhanced regeneration has been observed in the host brain using this therapeutic strategy (Wei et al., 2017).

## 5 Introducing the common types of stem cells for the treatment of ischemic stroke

### 5.1 Embryonic stem cells

Embryonic stem cells (ESCs), derived from the inner cell mass of the embryo before implantation, have unlimited self-renewal ability and the potential to differentiate into almost any cell type. ESCs could be differentiated into neural lineages using specific in vitro culture conditions (Bain et al., 1995). ESCs are identified as an optimal source of cell transplantation in order to treat neurological disorders. ESCs-derived cells which express cell surface markers of endothelial cells, astrocytes, neurons, and oligodendrocytes are observed in the lesion following transplantation of mouse ESCs into the cortex of mice suffering from ischemic stroke with subsequent functional recovery and structural improvement (Wei et al., 2005). Intrastriatal transplantation of ESCs-derived neuron-like cells or mouse ESCs was associated with improved dopaminergic function and consequently behavioral impairment in rates with focal ischemia with middle cerebral artery occlusion (MCAO) (Yanagisawa et al., 2006; Ye et al., 1998). Improved sensory and motor function of mice with MCAO have been observed following intracerebral transplantation of mouse ESCs which subsequently diminished infarct size.

Teratoma formation and malignant transformation are considered as disadvantages of ESC application. Limited resources, ethical issues, and high likelihood of malignant transformation are factors that limit the widespread ESC usage. There are very limited investigations on the applicability of ESCs in the treatment of stroke (Reubinoff et al., 2000). Malignant transformation of *in vivo* injected ESCs could be prevented using transplantation of differentiated cells derived from ESCs. Neural derivatives of ESCs are potential cells for the treatment of stroke. Numerous investigations have evaluated the impacts of ESC-derived neural stem/progenitor cells (NSPC) in the treatment of animal models of stroke (Daadi et al., 2008). Improved behavioral deficits, enhanced differentiation into neuronal cells, and diminished infarct area were observed following cell transplantation, regardless of the applied source of transplanted cells, the type of animal stroke model, and the kind of injection route. Meanwhile, numerous investigations have demonstrated the risk of teratoma formation using transplanted ESC-derived neurons (Sonntag et al., 2007). Culture conditions may reduce the risk of tumorigenesis of neural cells derived from transplanted ESCs. For example, an expandable and homogeneous population of NSCs called SD56 was isolated from ESCs using a medium supplemented with epidermal growth factor. After their implantation in the brain of ischemic rats, NSCs migrated to the parenchyma of the adult brain injured by ischemia. Besides, independent use of the stroke-impaired forelimb was improved two months following transplantation (Daadi et al., 2008). A higher density of cerebral blood vessels is parallel with a lower probability of stroke with a later occurrence. Any treatment which facilitates angiogenesis has a fundamental role in improving the performance of stroke patients. Intra-arterially transplanted human ESCs-derived endothelial and mural cells were associated with significantly increased vascular density and cerebral blood vessels in the ischemic striatum, diminished apoptosis and infarct volume, and facilitated neurological recovery in mice models of transient MCAO (Oyamada et al., 2008).

### 5.2 Inducible pluripotent stem cells

Inducible Pluripotent Stem Cells (iPSCs) are primarily induced from mouse adult fibroblasts or embryonic cells following transfection of four factors Sox2, Oct3/4, Klf4 and c-Myc (Takahashi and Yamanaka, 2006). These cells show the morphological and developmental characteristics of ESCs and express ESCs marker genes. *In vivo* subcutaneous transplantation of iPSCs into mice led to the development of tumors that contain diverse tissues derived from all three germinal layers (Tat et al., 2010). The advantage of iPSCs is the ability to proliferate and multipotent differentiation. Unlike ESCs, there is no ethical problem with using iPSCs. Chen et al. evaluated the therapeutic impacts of subdural transplantation of iPSCs combined with fibrin glue in mice with MCAO-induced ischemic stroke (S. J. Chen et al., 2010). Subdural transplantation of iPSCs has been demonstrated to diminish the total infarct volume. Diminished inflammatory responses in ischemic brain could be attributed to the protective and beneficial impacts of iPSCs. Other researchers have demonstrated migration of transplanted iPSCs derived from adult human fibroblasts into the damaged brain area with significant improvement of sensorimotor function in a rat model of MCAO (M. Jiang et al., 2011). However, behavioral improvement was not observed in one study using transplantation of iPSCs into mice brains with transient MCAO (Kawai et al., 2010). Differentiation of iPSCs into neurons and neuroblasts for ischemic stroke was observed representing a promising therapeutic strategy to supply sufficient neurons. The tumorigenic property is one of the concerns of iPSCs. Indeed, iPSCs have been demonstrated to form teratoma following transplantation into mouse ischemic brain (Yamashita et al., 2011). If tumorigenesis is properly controlled, iPSCs are potent candidates for the treatment of ischemic stroke.

### 5.3 Neural stem cells

Neural Stem Cells (NSCs) are present in the subventricular zone of the adult brain (Gage, 2000). After the onset of ischemic stroke, endogenous NSCs can proliferate and migrate to the damaged area and promote tissue repair (Yamashita et al., 2006). Endogenous NSCs contribute to post-ischemic brain repair. However, insufficient amount of endogenous NSCs in some instances results in improper replenishment of lost neurons, and a small number of NSCs differentiate into neurons (Arvidsson et al., 2002). Transplantation of NSCs can increase neurogenesis and is considered a promising therapeutic approach for ischemic stroke (Andres et al., 2011). Neuroprotection is induced with delay by intravenous NSC transplantation 3 days following ischemic stroke through suppression of inflammation and subsequent glial scar formation indicating the potential of NSCs for extension of the therapeutic window in the setting of ischemic stroke (Bacigaluppi et al., 2008). NSCs modified with Akt-1 or VEGF also improved neuronal function following ischemic stroke through enhancement of angiogenesis and neuronal survival (H. J. Lee et al., 2007). Therefore, NSCs are recognized as an effective candidate for the treatment of ischemic stroke.

### 5.4 Mesenchymal stem cells

**Fig. 1:**
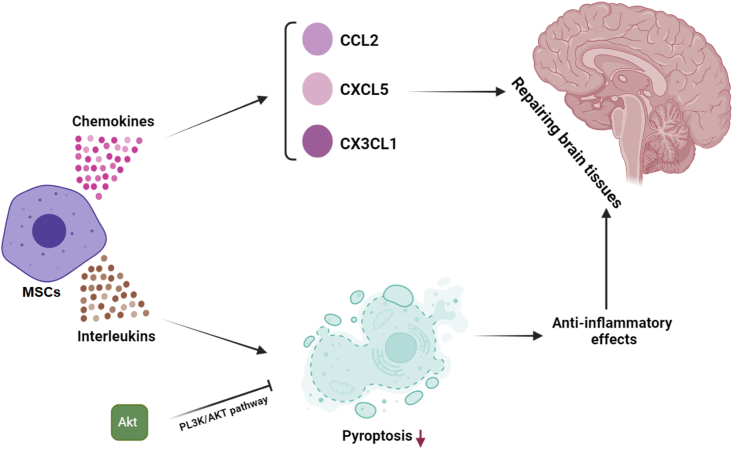
Therapeutic potential of mesenchymal stem cells in the ischemic brain. Cultured mesenchymal stem cells can release a variety of chemokines. These chemokines collaborate with mesenchymal stem cells to participate in improvement of damage or tissue repair of the ischemic brain. These cells can also release anti-inflammatory substances such as interleukins to regulate the operation of post-stroke immune system and play their anti-inflammatory role by inhibiting pyroptosis through the PL3K/AKT path.

The therapeutic capabilities of mesenchymal stem cells (MSCs) has been extensively investigated in the ischemic brain (Eckert et al., 2013). So far, it was not clear how transplanted cells contribute to improved functioning after ischemic stroke. Due to their limited neural differentiation capacity, MSCs have been found to exert their beneficial impacts principally through immunomodulatory and paracrine mechanisms rather than cell replacement (X. Liu et al., 2014). In vitro studies have demonstrated that the MSC environment significantly increased neurite outgrowth of dorsal root ganglion (Neuhuber et al., 2005). MSCs co-cultured with glutamate-exposed neurons significantly ameliorated neuronal damage induced by glutamate by releasing soluble neuroprotective factors (Hokari et al., 2008). Studies have shown that cultured mesenchymal stem cells can release a variety of chemokines. Cooperation of these chemokines with MSCs is essential for participation in the healing of ischemic brain tissue damage (Ringe et al., 2007). Mesenchymal stem cells can release anti-inflammatory substances such as interleukins (ILs) to regulate the function of the immune system after ischemic stroke, and play their anti-inflammatory role through the PL3K/AKT pathway by inhibiting pyroptosis (Fig. 1) (Walkowski et al., 2022; Zhou et al., 2019). *In vivo* investigations have demonstrated that MSC injection into mice following ischemic stroke was associated with reduced destruction of the blood-brain barrier and improved neurobehavioral recovery through inhibition of inflammation and induced angiogenesis and neurogenesis (J. Chen et al., 2003). *In vivo* transplantation of genetically engineered MSCs overexpressing IL-10 could significantly enhance mitophagy and autophagy with reduced markers of neuroinflammation and cell death compared with mere MSCs.

In stroke rat models, MSC transplantation was associated with enhanced axonal plasticity and interhemispheric and intracortical connections (Z. Liu et al., 2010). A number of investigations have recently demonstrated enhanced neurotrophic therapeutic impacts of MSCs by gene modification. For instance, intravenous injection of human MSCs modified with brain-derived neurotrophic factor (BDNF) and/or a combination of human MSCs with vascular endothelial growth factor and angiopoietin-1 into ischemic mice showed better therapeutic effects than yielded unmodified MSCs (Nomura et al., 2005).

### 5.5 Stem-cell-based gene therapy

In the last few years, a lot of experimental, preclinical and clinical data have been published showing the possibility of transferring new genetic information with relatively high efficiency in diverse cells or tissues such as differentiated cells and hematopoietic progenitor cells. Addition of normal gene to the cells with endogenous gene deletion, mutation, or alteration has been demonstrated experimentally to reverse phenotype and in some cases restore functional defect (Bagnis and Mannoni, 1997). Stem cell-based gene therapy is considered as a potential novel therapeutic strategy for the treatment of ischemic stroke in the future. Stem cells secrete different neurotrophic factors by themselves. Transplanted gene-modified stem cells overexpress various neurotrophic factors including noggin, BDNF, VEGF, HGF, GDNF, EPO, and NGF, and have been associated with significant improvement in functional recovery in the setting of stroke (Hao et al., 2014; H. J. Lee et al., 2007; H. Liu et al., 2006; Onda et al., 2008).

## 6 Clinical trials in the use of stem cells for the treatment of ischemic stroke

Stem cell-based therapies for the treatment of stroke opened promising horizons due to their ability to address patients' unmet requirements, neuroprotective and regenerative benefits (Mahla, 2016). The advantages of stem cell therapies for the treatment of stroke include neuroregeneration, reducing stroke-induced neuronal loss and neuroprotection, and functioning in the acute phase of stroke in order to restrict the spread of injury (F. Wang et al., 2018). Furthermore, although stroke is a vascular disease, a significant immune response is also present which correlates with the healing process. Certain populations of stem cells have the capacity for immune modulation, promising neuroprotective and neurodegenerative effects, and enhancement of therapeutic effects while reducing inflammatory damages (Caplan and Correa, 2011). Some clinical trials related to the treatment of stroke by stem cells are summarized in Table 1.

**Table 1:** Clinical Trials in the treatment of stroke using stem cells.

Stem cell type	Features	Outcomes	Reference
MSC	In South Korea with 54 subjects	Intravenous infusion of preconditioned, autologous MSCs accompanied with autologous serum was observed to be both safe and feasible in cases afflicted with chronic stroke. No improvement was seen in the 3-month mRS score following treatment with MSC, while they observed improvement in leg motor manifested by detailed functional analyses.	Chung et al. (2021)
MSC	In South Korea with 30 subjects	Intravenous application of autologous MSCs was identified as a safe and feasible therapeutic approach in cases with significant cerebral infarcts which possibly be associated with functional recovery.	Bang et al. (2005)
MSC	In South Korea with 54 subjects	Stem cell-based therapeutic approaches are able to facilitate motor recovery following stroke by enhancement of protection of corticospinal tract against degeneration and positive alterations in network recognition.	(J. Lee et al., 2022)
MSC	Phase II, in Spain with 19 subjects	Total adverse events (AEs) numbers and neurologic and systemic complications were similar between groups. No injection-related AEs and or tumor development were registered. Patients in adipose tissue-derived- mesenchymal stem cells (AD-MSCs) groups demonstrated a non-significantly lower median NIHSS score within 24 months of follow-up. Besides, no differences were observed between groups regarding mRS scores up to 24 months of follow-up. Thus, intravenous treatment using AD-MSCs during the first two weeks of ischemic stroke was demonstrated to be safe up to 24 months of follow-up.	de Celis-Ruiz et al. (2022)
MSCs	Phase II, in Netherlands with 10 subjects, cell dose: 45-50 × 10^6^	This first-in-human investigation revealed feasibility of intranasal application of bone marrow-derived MSC in neonates following Perinatal arterial ischemic stroke (PAIS) without any serious adverse events until 3 months of age. Future large-scale placebo-controlled investigations are required to evaluate the therapeutic benefits of intranasal MSCs in the setting of PAIS.	Baak et al. (2022)
MSCs	In South Korea with 54 subjects	This is the first trial demonstrating the therapeutic advantages of MSCs in the setting of ischemic stroke with significant enhancement in circulating extracellular vesicles (EVs) and significant correlation with improvement of motor function and indices of plasticity based on magnetic resonance imaging.	Bang et al. (2022)
MSCs	Phase I, in France, with 31 subjects	Intravenous infusion of autologous MSCs following stroke was shown to be both safe and feasible. The results of task-related motor cortex (MI) activity and motor performance suggest improvement of motor recovery through sensorimotor neuroplasticity using MSCs.	Jaillard et al. (2020)
MSC	Phase II, in six European Countries with 138 subjects	Clinical data regarding safety and efficacy of intra-myocardial application of allogeneic adipose-derived stromal cells in cases with Ischemic heart failure (IHF) derived from healthy donors will be provided by the Stem Cell therapy in IschEmic Non-treatable Cardiac diseasE (SCIENCE) trial.	Paitazoglou et al. (2019)
MSC	Phase I, in USA with 10 subjects	Despite the inherent limitations of phase I clinical trial, the results of this study demonstrates the safety and feasibility of intracoronary infusion of combined cell products and implies to the benefits of this type of therapy.	Lasala et al. (2011)
MSCs	In South Korea with 52 subjects	Intravenous infusion of autologous MSCs was demonstrated to be safe for stroke cases during long-term follow-up period. This therapeutic strategy might improve functional recovery following stroke based on specific patient characteristics.	(J. S. Lee et al., 2010)
MSCs	In China with 8 subjects, cell dose: 0.5 × 10^6^/kg body weight	The disability levels, neurological functions, and daily living abilities of participants in this study were all improved. Although these observations indicate the safety and feasibility of the applied combination of transplanted neural stem/progenitor cells (NSPCs) and MSCs for the improvement of neurological function, further investigations with larger sample volume studies including larger samples, longer follow-up periods and control groups are required.	Qiao et al. (2014)
MSC	In China with 4 subjects, cell dose: 2 × 10^7^	No improvement in muscle strength was observed in hemorrhagic stroke cases and patients did not amend their daily activities. Intra-artery infusion of UCMSCs through catheterization was demonstrated to be a safe and feasible approach for the improvement of neurological functions of cases with ischemic stroke in the setting of infarction in the middle cerebral artery territory.	(Y. Jiang et al., 2013)
MSC	In South Korea with 60 subjects	This is the first trial on evaluation of the efficacy of MSCs in cases with ischemic stroke. The results could provide more evidence regarding the efficacy of MSCs in cases with ischemic stroke.	Kim et al. (2013)
MSC	In Miami with 13 subjects	Although the scar size was diminished using both cell doses, ejection fraction was only enhanced using 100 million doses which highlights the fundamental role of cell dose in effectiveness of cell therapy. Identification of optimal dosage and delivery method is crucial for advancement of the field, illustration of mechanisms of action and enhancement of planning for phase III trials.	Florea et al. (2017)
MSC	Phase II, In Malaysia with 34 subjects	Intravenous administration of BMMSCs in cases with subacute MCA infarction was demonstrated to be both safe and well tolerated. However, no improvement was observed in functional outcomes of neurological recovery following 12 months. Meanwhile, median infarct volume was improved in treatment group. Certainly, larger studies are required to confirm the efficacy of BMMSCs in ischemic strike cases.	Law et al. (2021)

## 7 Challenges of ischemic stroke treatment with stem cell therapy

Many hurdles must be overcome before successful application of stem cells in the clinic including the strategy of tracking transplanted cells and limited life. *In vivo* cell tracking is applicable through positron emission tomography (PET), single-photon emission computed tomography (SPECT/CT), bioluminescence imaging (BLI) using GFP protein, and magnetic resonance imaging (MRI) (Zheng et al., 2017). Moshayedi et al. demonstrated enhanced survival of transplanted cells using hyaluronic acid (HA) hydrogels for at least 6 weeks and tracked them *in vivo* using MRI (Moshayedi et al., 2016). Besides, there are several ethical concerns regarding clinical application of stem cells, mainly with ESCs and NSCs. Application of iPSCs might avoid this problem. In addition, production of autologous iPSCs is possible, but relatively expensive and takes several months to prepare the cells for transplantation. In addition, the clinical usage of stem cells might raise several safety concerns. For example, allograft transplantation might result in immune rejection. Potentially, stem cells could differentiate into undesirable tissues which enhance tumor growth and its metastasis by increasing the production of neo-vessels and altering of tumor microenvironment (Patel et al., 2010). Erdö et al. showed increased tumorigenesis by contamination of undifferentiated Embryonic Stem Cells (Erdö et al., 2003). Amariglio et al. reported the development of brain tumors four years following the initial treatment in cases with ataxia-telangiectasia who were treated with intraspinal and intracerebellar injection of donor-derived neural stem cells (Amariglio et al., 2009). More clinical research is necessary to discover the optimal routes of transplantation, such as the appropriate time, route and dose of injection.

## 8 Conclusion and prospective

After the damage induced by ischemic stroke, the application of methods that improve the tissue conditions and reduce the destructive effects is required. Since the general process of ischemic damage is very complicated and the pattern of pathophysiological changes is not the same for all brain cells, a proper understanding of the cellular and molecular alterations caused by cerebral ischemia is necessary to create strategies for the treatment of cerebral stroke. Due to the surgical hazards and the challenges of using chemical drugs for the treatment of stroke, they cannot be considered as safe approach for the treatment of ischemic stroke. On the other hand, stem cells are an attractive candidate for the treatment of ischemic stroke. The beneficial impacts of stem cells include synaptogenesis, neuroprotection, angiogenesis, inflammation, immune responses, and others. However, before clinical application, many important issues including optimal cell sources, dosage, timing, and monitoring of events and side effects must be managed. A better understanding of the mechanisms of action of stem cells in the treating ischemic stroke helps to solve the above problems. Considering the rapid progress of gene therapy and the widespread use of stem cells in this field, stem cells along with gene therapy in future experiments and clinical programs can play an important role in the treatment of cerebral ischemia. Despite the lingering uncertainties surrounding potential side effects, ongoing research suggests a promising role for stem cell therapy in treating these patients. However, further evidence is required to fully establish its effectiveness and safety.

## Ethics statement

Not applicable.

## Funding

Non.

## CRediT authorship contribution statement

**Sahar Yaqubi:** prepared figures, contributed to the drafting of the text, All authors reviewed the manuscript. **Mohammad Karimian:** contributed to the conception and design of the study, prepared figures, contributed to the drafting of the text.

## Declaration of competing interest

There is no conflict of interest.

## Data Availability

No data was used for the research described in the article.

## References

[bib1] Abubakar M., Masood M.F., Javed I., Adil H., Faraz M.A., Bhat R.R., Hajjaj M. (2023). Unlocking the mysteries, bridging the gap, and unveiling the multifaceted potential of stem cell therapy for cardiac tissue regeneration: a narrative review of current literature, ethical challenges, and future perspectives. Cureus.

[bib2] Aleksic D.Z., Jankovic S.M., Mlosavljevic M.N., Toncev G.L., Miletic Drakulic S.D., Stefanovic S.M. (2019). Potential drug-drug interactions in acute ischemic stroke patients at the neurological intensive care unit. Open Med..

[bib3] Amariglio N., Hirshberg A., Scheithauer B.W., Cohen Y., Loewenthal R., Trakhtenbrot L., Rechavi G. (2009). Donor-derived brain tumor following neural stem cell transplantation in an ataxia telangiectasia patient. PLoS Med..

[bib4] Andres R.H., Horie N., Slikker W., Keren-Gill H., Zhan K., Sun G., Steinberg G.K. (2011). Human neural stem cells enhance structural plasticity and axonal transport in the ischaemic brain. Brain.

[bib5] Arvidsson A., Collin T., Kirik D., Kokaia Z., Lindvall O. (2002). Neuronal replacement from endogenous precursors in the adult brain after stroke. Nat Med.

[bib6] Baak L.M., Wagenaar N., van der Aa N.E., Groenendaal F., Dudink J., Tataranno M.L., Benders M. (2022). Feasibility and safety of intranasally administered mesenchymal stromal cells after perinatal arterial ischaemic stroke in The Netherlands (PASSIoN): a first-in-human, open-label intervention study. Lancet Neurol..

[bib7] Bacigaluppi M., Pluchino S., Martino G., Kilic E., Hermann D.M. (2008). Neural stem/precursor cells for the treatment of ischemic stroke. J. Neurol. Sci..

[bib8] Bagnis C., Mannoni P. (1997). Stem cell-based gene therapy. Oncol..

[bib9] Bain G., Kitchens D., Yao M., Huettner J.E., Gottlieb D.I. (1995). Embryonic stem cells express neuronal properties in vitro. Dev. Biol..

[bib10] Bang O.Y., Kim E.H., Cho Y.H., Oh M.J., Chung J.W., Chang W.H., Chopp M. (2022). Circulating extracellular vesicles in stroke patients treated with mesenchymal stem cells: a biomarker analysis of a randomized trial. Stroke.

[bib11] Bang O.Y., Lee J.S., Lee P.H., Lee G. (2005). Autologous mesenchymal stem cell transplantation in stroke patients. Ann. Neurol..

[bib12] Bansal S., Sangha K.S., Khatri P. (2013). Drug treatment of acute ischemic stroke. Am. J. Cardiovasc. Drugs.

[bib13] Barahimi P., Karimian M., Nejati M., Azami Tameh A., Atlasi M.A. (2021). Oxytocin improves ischemic stroke by reducing expression of excitatory amino acid transporter 3 in rat MCAO model. J. Immunoassay Immunochem..

[bib14] Bath P.M., Sprigg N. (2006). Colony stimulating factors (including erythropoietin, granulocyte colony stimulating factor and analogues) for stroke. Cochrane Database Syst. Rev..

[bib15] Behdarvandy M., Karimian M., Atlasi M.A., Azami Tameh A. (2020). Heat shock protein 27 as a neuroprotective biomarker and a suitable target for stem cell therapy and pharmacotherapy in ischemic stroke. Cell Biol. Int..

[bib16] Berkhemer O.A., Fransen P.S., Beumer D., van den Berg L.A., Lingsma H.F., Yoo A.J., Dippel D.W. (2015). A randomized trial of intraarterial treatment for acute ischemic stroke. N. Engl. J. Med..

[bib17] Boehme A.K., Esenwa C., Elkind M.S. (2017). Stroke risk factors, genetics, and prevention. Circ. Res..

[bib18] Brzica H., Abdullahi W., Ibbotson K., Ronaldson P.T. (2017). Role of transporters in central nervous system drug delivery and blood-brain barrier protection: relevance to treatment of stroke. J. Cent. Nerv. Syst. Dis..

[bib19] Caplan A.I. (2007). Adult mesenchymal stem cells for tissue engineering versus regenerative medicine. J. Cell. Physiol..

[bib20] Caplan A.I., Correa D. (2011). The MSC: an injury drugstore. Cell Stem Cell.

[bib21] Chen J., Zhang Z.G., Li Y., Wang L., Xu Y.X., Gautam S.C., Chopp M. (2003). Intravenous administration of human bone marrow stromal cells induces angiogenesis in the ischemic boundary zone after stroke in rats. Circ. Res..

[bib22] Chen S.J., Chang C.M., Tsai S.K., Chang Y.L., Chou S.J., Huang S.S., Chiou S.H. (2010). Functional improvement of focal cerebral ischemia injury by subdural transplantation of induced pluripotent stem cells with fibrin glue. Stem Cells Dev.

[bib23] Chung J.W., Chang W.H., Bang O.Y., Moon G.J., Kim S.J., Kim S.K., Kim Y.H. (2021). Efficacy and safety of intravenous mesenchymal stem cells for ischemic stroke. Neurology.

[bib24] Daadi M.M., Maag A.L., Steinberg G.K. (2008). Adherent self-renewable human embryonic stem cell-derived neural stem cell line: functional engraftment in experimental stroke model. PLoS One.

[bib25] de Celis-Ruiz E., Fuentes B., Alonso de Leciñana M., Gutiérrez-Fernández M., Borobia A.M., Gutiérrez-Zúñiga R., Díez-Tejedor E. (2022). Final results of allogeneic adipose tissue-derived mesenchymal stem cells in acute ischemic stroke (AMASCIS): a phase II, randomized, double-blind, placebo-controlled, single-center, pilot clinical trial. Cell Transplant..

[bib26] Eckert M.A., Vu Q., Xie K., Yu J., Liao W., Cramer S.C., Zhao W. (2013). Evidence for high translational potential of mesenchymal stromal cell therapy to improve recovery from ischemic stroke. J Cereb Blood Flow Metab.

[bib27] Erdö F., Bührle C., Blunk J., Hoehn M., Xia Y., Fleischmann B., Trapp T. (2003). Host-dependent tumorigenesis of embryonic stem cell transplantation in experimental stroke. J Cereb Blood Flow Metab.

[bib28] Falkenburg J.H., Heslop H.E., Barrett A.J. (2008). T cell therapy in allogeneic stem cell transplantation. Biol. Blood Marrow Transplant..

[bib29] Florea V., Rieger A.C., DiFede D.L., El-Khorazaty J., Natsumeda M., Banerjee M.N., Hare J.M. (2017). Dose comparison study of allogeneic mesenchymal stem cells in patients with ischemic cardiomyopathy (the TRIDENT study). Circ. Res..

[bib30] Gage F.H. (2000). Mammalian neural stem cells. Science.

[bib31] Gallichio J.E. (2004). Pharmacologic management of spasticity following stroke. Phys. Ther..

[bib32] George B. (2011). Regulations and guidelines governing stem cell based products: clinical considerations. Perspect Clin Res.

[bib33] Gilman S. (2006). Pharmacologic management of ischemic stroke: relevance to stem cell therapy. Exp. Neurol..

[bib34] Hao L., Zou Z., Tian H., Zhang Y., Zhou H., Liu L. (2014). Stem cell-based therapies for ischemic stroke. BioMed Res. Int..

[bib35] Hess D.C., Borlongan C.V. (2008). Cell-based therapy in ischemic stroke. Expert Rev. Neurother..

[bib36] Hinkle J.L., Guanci M.M. (2007). Acute ischemic stroke review. J. Neurosci. Nurs..

[bib37] Hochain P., Capet C., Colin R. (2000). [Digestive complications of aspirin]. Rev. Med. Interne.

[bib38] Hokari M., Kuroda S., Shichinohe H., Yano S., Hida K., Iwasaki Y. (2008). Bone marrow stromal cells protect and repair damaged neurons through multiple mechanisms. J. Neurosci. Res..

[bib39] Jaillard A., Hommel M., Moisan A., Zeffiro T.A., Favre-Wiki I.M., Barbieux-Guillot M., Detante O. (2020). Autologous mesenchymal stem cells improve motor recovery in subacute ischemic stroke: a randomized clinical trial. Transl Stroke Res.

[bib40] Jiang M., Lv L., Ji H., Yang X., Zhu W., Cai L., Dong Q. (2011). Induction of pluripotent stem cells transplantation therapy for ischemic stroke. Mol. Cell. Biochem..

[bib41] Jiang Y., Zhu W., Zhu J., Wu L., Xu G., Liu X. (2013). Feasibility of delivering mesenchymal stem cells via catheter to the proximal end of the lesion artery in patients with stroke in the territory of the middle cerebral artery. Cell Transplant..

[bib42] Jin Y., Li S., Yu Q., Chen T., Liu D. (2023). Application of stem cells in regeneration medicine. MedComm.

[bib43] Kawai H., Yamashita T., Ohta Y., Deguchi K., Nagotani S., Zhang X., Abe K. (2010). Tridermal tumorigenesis of induced pluripotent stem cells transplanted in ischemic brain. J Cereb Blood Flow Metab.

[bib44] Khassafi N., Zahraei Z., Vahidinia Z., Karimian M., Azami Tameh A. (2022). Calcitriol pretreatment attenuates glutamate neurotoxicity by regulating NMDAR and CYP46A1 gene expression in rats subjected to transient middle cerebral artery occlusion. J. Neuropathol. Exp. Neurol..

[bib45] Khatri P., Abruzzo T., Yeatts S.D., Nichols C., Broderick J.P., Tomsick T.A. (2009). Good clinical outcome after ischemic stroke with successful revascularization is time-dependent. Neurology.

[bib46] Kikuchi K., Tanaka E., Murai Y., Tancharoen S. (2014). Clinical trials in acute ischemic stroke. CNS Drugs.

[bib47] Kim S.J., Moon G.J., Chang W.H., Kim Y.H., Bang O.Y. (2013). Intravenous transplantation of mesenchymal stem cells preconditioned with early phase stroke serum: current evidence and study protocol for a randomized trial. Trials.

[bib48] Kuriakose D., Xiao Z. (2020). Pathophysiology and treatment of stroke: present status and future perspectives. Int. J. Mol. Sci..

[bib49] Lasala G.P., Silva J.A., Kusnick B.A., Minguell J.J. (2011). Combination stem cell therapy for the treatment of medically refractory coronary ischemia: a Phase I study. Cardiovasc Revasc Med.

[bib50] Law Z.K., Tan H.J., Chin S.P., Wong C.Y., Wan Yahya W.N.N., Muda A.S., Mohamed Ibrahim N. (2021). The effects of intravenous infusion of autologous mesenchymal stromal cells in patients with subacute middle cerebral artery infarct: a phase 2 randomized controlled trial on safety, tolerability and efficacy. Cytotherapy.

[bib51] Leader B., Baca Q.J., Golan D.E. (2008). Protein therapeutics: a summary and pharmacological classification. Nat. Rev. Drug Discov..

[bib52] Lee H.J., Kim K.S., Park I.H., Kim S.U. (2007). Human neural stem cells over-expressing VEGF provide neuroprotection, angiogenesis and functional recovery in mouse stroke model. PLoS One.

[bib53] Lee J., Chang W.H., Chung J.W., Kim S.J., Kim S.K., Lee J.S., Bang O.Y. (2022). Efficacy of intravenous mesenchymal stem cells for motor recovery after ischemic stroke: a neuroimaging study. Stroke.

[bib54] Lee J.S., Hong J.M., Moon G.J., Lee P.H., Ahn Y.H., Bang O.Y. (2010). A long-term follow-up study of intravenous autologous mesenchymal stem cell transplantation in patients with ischemic stroke. Stem Cell..

[bib55] Levy E.I., Boulos A.S., Fessler R.D., Bendok B.R., Ringer A.J., Kim S.H., Hopkins L.N. (2002). Transradial cerebral angiography: an alternative route. Neurosurgery.

[bib56] Liu H., Honmou O., Harada K., Nakamura K., Houkin K., Hamada H., Kocsis J. (2006). Neuroprotection by PlGF gene-modified human mesenchymal stem cells after cerebral ischaemia. Brain.

[bib57] Liu X., Ye R., Yan T., Yu S.P., Wei L., Xu G., Chen J. (2014). Cell based therapies for ischemic stroke: from basic science to bedside. Prog Neurobiol.

[bib58] Liu Z., Li Y., Zhang Z.G., Cui X., Cui Y., Lu M., Chopp M. (2010). Bone marrow stromal cells enhance inter- and intracortical axonal connections after ischemic stroke in adult rats. J Cereb Blood Flow Metab.

[bib59] Louria D.B. (1969). Outpatient service and community health centers. Bull N J Coll Med Dent.

[bib60] Mahla R.S. (2016). Stem cells applications in regenerative medicine and disease therapeutics. Int J Cell Biol.

[bib61] Mansour A., Romani M., Acharya A.B., Rahman B., Verron E., Badran Z. (2023). Drug delivery systems in regenerative medicine: an updated review. Pharmaceutics.

[bib62] Marei H.E., Hasan A., Rizzi R., Althani A., Afifi N., Cenciarelli C., Shuaib A. (2018). Potential of stem cell-based therapy for ischemic stroke. Front. Neurol..

[bib63] Misra V., Ritchie M.M., Stone L.L., Low W.C., Janardhan V. (2012). Stem cell therapy in ischemic stroke: role of IV and intra-arterial therapy. Neurology.

[bib64] Moshayedi P., Nih L.R., Llorente I.L., Berg A.R., Cinkornpumin J., Lowry W.E., Carmichael S.T. (2016). Systematic optimization of an engineered hydrogel allows for selective control of human neural stem cell survival and differentiation after transplantation in the stroke brain. Biomaterials.

[bib65] Nejati M., Atlasi M.A., Karimian M., Nikzad H., Azami Tameh A. (2018). Lipoprotein lipase gene polymorphisms as risk factors for stroke: a computational and meta-analysis. Iran J Basic Med Sci.

[bib66] Neuhuber B., Timothy Himes B., Shumsky J.S., Gallo G., Fischer I. (2005). Axon growth and recovery of function supported by human bone marrow stromal cells in the injured spinal cord exhibit donor variations. Brain Res..

[bib67] Nomura T., Honmou O., Harada K., Houkin K., Hamada H., Kocsis J.D. (2005). I.V. infusion of brain-derived neurotrophic factor gene-modified human mesenchymal stem cells protects against injury in a cerebral ischemia model in adult rat. Neuroscience.

[bib68] Nozohouri S., Sifat A.E., Vaidya B., Abbruscato T.J. (2020). Novel approaches for the delivery of therapeutics in ischemic stroke. Drug Discov. Today.

[bib69] Onda T., Honmou O., Harada K., Houkin K., Hamada H., Kocsis J.D. (2008). Therapeutic benefits by human mesenchymal stem cells (hMSCs) and Ang-1 gene-modified hMSCs after cerebral ischemia. J. Cerebr. Blood Flow Metabol..

[bib70] Oyamada N., Itoh H., Sone M., Yamahara K., Miyashita K., Park K., Nakao K. (2008). Transplantation of vascular cells derived from human embryonic stem cells contributes to vascular regeneration after stroke in mice. J. Transl. Med..

[bib71] Paitazoglou C., Bergmann M.W., Vrtovec B., Chamuleau S.A.J., van Klarenbosch B., Wojakowski W., Kastrup J. (2019). Rationale and design of the European multicentre study on stem cell therapy in IschEmic non-treatable cardiac diseasE (SCIENCE). Eur. J. Heart Fail..

[bib72] Pardridge W.M. (2001). Brain drug targeting and gene technologies. Jpn. J. Pharmacol..

[bib73] Patel S.A., Meyer J.R., Greco S.J., Corcoran K.E., Bryan M., Rameshwar P. (2010). Mesenchymal stem cells protect breast cancer cells through regulatory T cells: role of mesenchymal stem cell-derived TGF-beta. J. Immunol..

[bib74] Qiao L.Y., Huang F.J., Zhao M., Xie J.H., Shi J., Wang J., Geng T.C. (2014). A two-year follow-up study of cotransplantation with neural stem/progenitor cells and mesenchymal stromal cells in ischemic stroke patients. Cell Transplant..

[bib75] Reubinoff B.E., Pera M.F., Fong C.Y., Trounson A., Bongso A. (2000). Embryonic stem cell lines from human blastocysts: somatic differentiation in vitro. Nat. Biotechnol..

[bib76] Ringe J., Strassburg S., Neumann K., Endres M., Notter M., Burmester G.R., Sittinger M. (2007). Towards in situ tissue repair: human mesenchymal stem cells express chemokine receptors CXCR1, CXCR2 and CCR2, and migrate upon stimulation with CXCL8 but not CCL2. J. Cell. Biochem..

[bib77] Rosen M.R. (2006). Are stem cells drugs? The regulation of stem cell research and development. Circulation.

[bib78] Rubin L.L., Haston K.M. (2011). Stem cell biology and drug discovery. BMC Biol..

[bib79] Sonntag K.C., Pruszak J., Yoshizaki T., van Arensbergen J., Sanchez-Pernaute R., Isacson O. (2007). Enhanced yield of neuroepithelial precursors and midbrain-like dopaminergic neurons from human embryonic stem cells using the bone morphogenic protein antagonist noggin. Stem Cell..

[bib80] Tajalli-Nezhad S., Karimian M., Beyer C., Atlasi M.A., Azami Tameh A. (2019). The regulatory role of Toll-like receptors after ischemic stroke: neurosteroids as TLR modulators with the focus on TLR2/4. Cell. Mol. Life Sci..

[bib81] Takahashi K., Yamanaka S. (2006). Induction of pluripotent stem cells from mouse embryonic and adult fibroblast cultures by defined factors. Cell.

[bib82] Tameh A.A., Karimian M., Zare-Dehghanani Z., Aftabi Y., Beyer C. (2018). Role of steroid therapy after ischemic stroke by n-Methyl-d-Aspartate receptor gene regulation. J. Stroke Cerebrovasc. Dis..

[bib83] Tat P.A., Sumer H., Jones K.L., Upton K., Verma P.J. (2010). The efficient generation of induced pluripotent stem (iPS) cells from adult mouse adipose tissue-derived and neural stem cells. Cell Transplant..

[bib84] Tremonti C., Thieben M. (2021). Drugs in secondary stroke prevention. Aust. Prescr..

[bib85] Vahidinia Z., Joghataei M.T., Beyer C., Karimian M., Tameh A.A. (2021). G-Protein-Coupled receptors and ischemic stroke: a focus on molecular function and therapeutic potential. Mol. Neurobiol..

[bib86] Vahidinia Z., Karimian M., Joghataei M.T. (2020). Neurosteroids and their receptors in ischemic stroke: from molecular mechanisms to therapeutic opportunities. Pharmacol. Res..

[bib87] Vahidinia Z., Khassafi N., Tameh A.A., Karimian M., Zare-Dehghanani Z., Moradi F., Joghataei M.T. (2022). Calcitriol ameliorates brain injury in the rat model of cerebral ischemia-reperfusion through Nrf2/HO-1 signalling Axis: an in silico and in vivo study. J. Stroke Cerebrovasc. Dis..

[bib88] Van Pham P. (2016). Stem cell drugs: the next generation of pharmaceutical products. Biomedical Research and Therapy.

[bib89] Walkowski B., Kleibert M., Majka M., Wojciechowska M. (2022). Insight into the role of the PI3K/Akt pathway in ischemic injury and post-infarct left ventricular remodeling in normal and diabetic heart. Cells.

[bib90] Wang F., Tang H., Zhu J., Zhang J.H. (2018). Transplanting mesenchymal stem cells for treatment of ischemic stroke. Cell Transplant..

[bib91] Wang J., Deng G., Wang S., Li S., Song P., Lin K., He Z. (2024). Enhancing regenerative medicine: the crucial role of stem cell therapy. Front. Neurosci..

[bib92] Wei L., Cui L., Snider B.J., Rivkin M., Yu S.S., Lee C.S., Choi D.W. (2005). Transplantation of embryonic stem cells overexpressing Bcl-2 promotes functional recovery after transient cerebral ischemia. Neurobiol. Dis..

[bib93] Wei L., Wei Z.Z., Jiang M.Q., Mohamad O., Yu S.P. (2017). Stem cell transplantation therapy for multifaceted therapeutic benefits after stroke. Prog Neurobiol.

[bib94] Wittich C.M., Burkle C.M., Lanier W.L. (2012). Ten common questions (and their answers) about off-label drug use. Mayo Clin. Proc..

[bib95] Yamashita T., Kawai H., Tian F., Ohta Y., Abe K. (2011). Tumorigenic development of induced pluripotent stem cells in ischemic mouse brain. Cell Transplant..

[bib96] Yamashita T., Ninomiya M., Hernández Acosta P., García-Verdugo J.M., Sunabori T., Sakaguchi M., Sawamoto K. (2006). Subventricular zone-derived neuroblasts migrate and differentiate into mature neurons in the post-stroke adult striatum. J. Neurosci..

[bib97] Yanagisawa D., Qi M., Kim D.H., Kitamura Y., Inden M., Tsuchiya D., Inoue K. (2006). Improvement of focal ischemia-induced rat dopaminergic dysfunction by striatal transplantation of mouse embryonic stem cells. Neurosci. Lett..

[bib98] Ye W., Shimamura K., Rubenstein J.L., Hynes M.A., Rosenthal A. (1998). FGF and Shh signals control dopaminergic and serotonergic cell fate in the anterior neural plate. Cell.

[bib99] Zheng Y., Huang J., Zhu T., Li R., Wang Z., Ma F., Zhu J. (2017). Stem cell tracking technologies for neurological regenerative medicine purposes. Stem Cells Int.

[bib100] Zhou T., Sun Y., Wang Y., Chen X., Zhuo L., Bu L., Shi J. (2019). Umbilical cord blood mesenchymal stem cells enhance lipopolysaccharide-induced IL-10 and IL-37 production in THP-1 cells. Inflammation.

[bib101] Zhu S.Z., Szeto V., Bao M.H., Sun H.S., Feng Z.P. (2018). Pharmacological approaches promoting stem cell-based therapy following ischemic stroke insults. Acta Pharmacol. Sin..

